# High-dose stereotactic radiotherapy boost in the treatment of squamous cell carcinoma of the head and neck region

**DOI:** 10.1186/s13014-022-02069-4

**Published:** 2022-06-03

**Authors:** Paweł Polanowski, Agnieszka Pietruszka, Dorota Księżniak-Baran, Aleksandra Grządziel, Ewa Chmielik, Marek Kentnowski, Natalia Amrogowicz, Krzysztof Składowski, Katarzyna Polanowska

**Affiliations:** 1 1St Radiation and Clinical Oncology Department, Maria Sklodowska-Curie National Research Institute of Oncology, Gliwice Branch, Gliwice, Poland; 2 Radiotherapy Planning Department, Maria Sklodowska-Curie National Research Institute of Oncology, Gliwice Branch, Gliwice, Poland; 3 Tumor Pathology Department, Maria Sklodowska-Curie National Research Institute of Oncology, Gliwice Branch, Gliwice, Poland; 4 Ophthalmology Department, St. Barbara Provincial Hospital No 5, Sosnowiec, Poland

**Keywords:** Squamous cell carcinoma, Stereotactic radiotherapy, Radiosurgery boost, Head and neck cancer

## Abstract

**Background:**

Surgical resection with adjuvant concurrent radiochemotherapy is the standard of care for stage III–IV oral cavity cancer. In some cases, the dynamic course of the disease is out of the prepared schedule of treatment. In that event, a stereotactic radiosurgery boost might be the only chance for disease control.

**Case presentation:**

Here, we present a case study of a patient with oral cancer who underwent surgery. During adjuvant radiotherapy, a metastatic cervical lymph node was diagnosed based on fine-needle aspiration biopsy. To increase the total dose to the metastatic tumor, a stereotactic radiosurgery boost of 1 × 18 Gy was performed two days after the last fraction of conventional radiotherapy. The early and late tolerance of this treatment were positive. During the 18-month follow-up, locoregional recurrence was not detected. The patient died due to secondary malignancy.

**Conclusions:**

This paper shows that a stereotactic radiosurgery boost added to adjuvant conventional radiotherapy is an effective approach permitting the maintenance of good local control in well-selected patients.

## Introduction

Squamous cell carcinoma is the most common malignant tumor in the head and neck anatomical region. The lip and oral cavity are the predominant sites of head and neck carcinoma according to the Globocan database in terms of morbidity and mortality [1]. A surgical procedure involving tumorectomy and lymphadenectomy, followed by radiotherapy (RTH) or radiochemotherapy, is the standard of treatment [2, 3]. Unfortunately, in some cases, disease progression occurs in a short period of time after the operation during adjuvant treatment. Reoperation is one of the options for proceeding, but the constricted healing process caused by radiation-induced acute side effects limits the possibility of its safe execution. Another option is the attempted use of an unconventional scheme of treatment, such as increasing the total dose of radiation. In this paper, we describe how the application of a high-dose boost can avoid a reoperation procedure and achieve a complete response and good local control (LC).

## Case report

We present a 43-year-old nonsmoking and nondrinking white male with oral cancer on the left side of the tongue. A biopsy sample taken from the tumor revealed a G2 keratinizing squamous cell carcinoma. On a CT scan performed two months before the operation, the radiologist described a primary tumor measuring 15 × 10 mm with peripheral contrast enhancement and two suspected, probably metastatic lymph nodes on the left side of the neck in groups III and IV, size 17 × 14 mm and 26 × 16 mm, respectively. Distant metastases were ruled out on chest X-ray and abdominal ultrasound. Moreover, anamnesis proved previous lower extremity deep vein thrombosis, but Doppler ultrasound ruled out active disease.

**Fig. 1:**
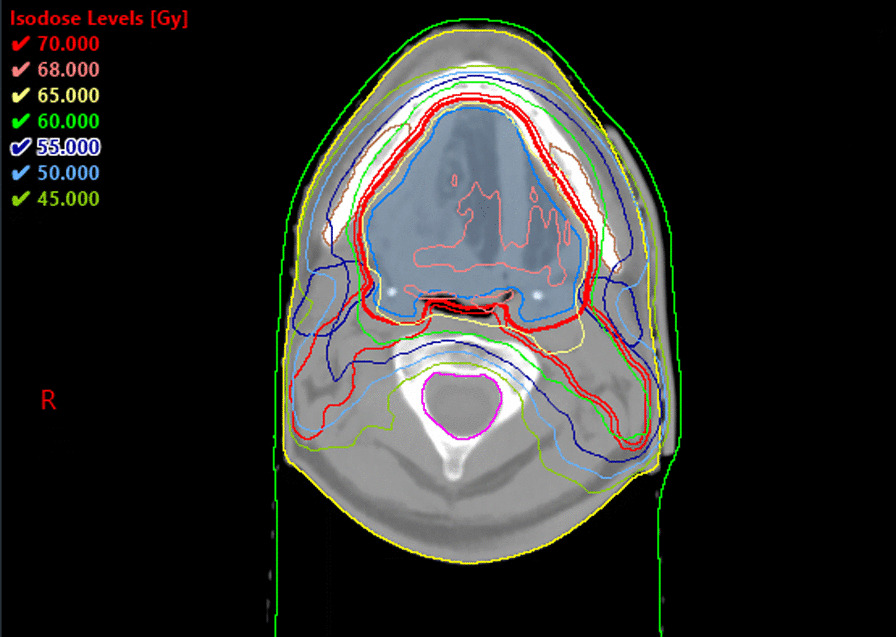
Conventional radiotherapy. Dose distribution shown on the CT scan. Isocenter plane of the 3rd stage of initial plan

**Fig. 2:**
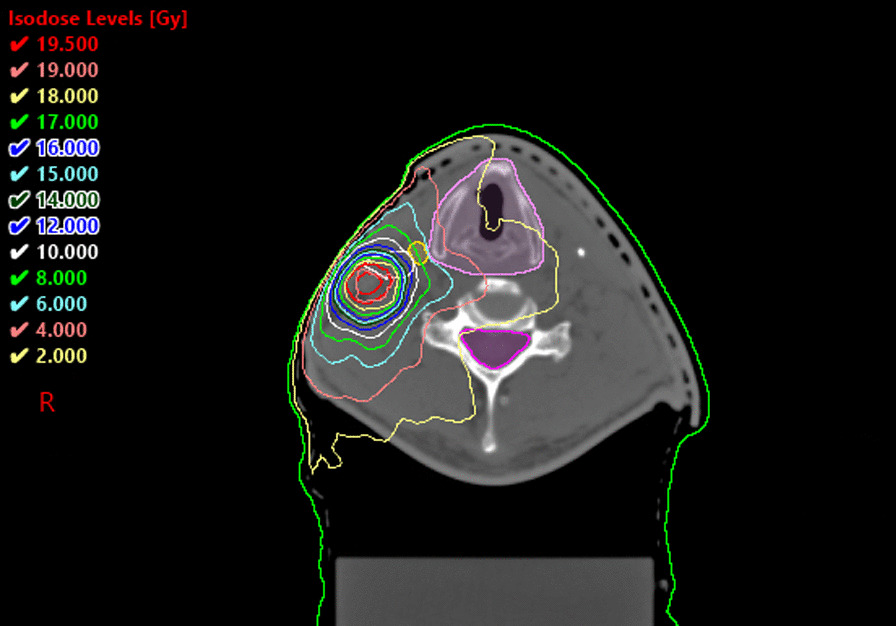
Stereotactic radiosurgery boost. Dose distribution shown on the CT scan. Isocenter plane of the stereotactic boost plan

**Fig. 3:**
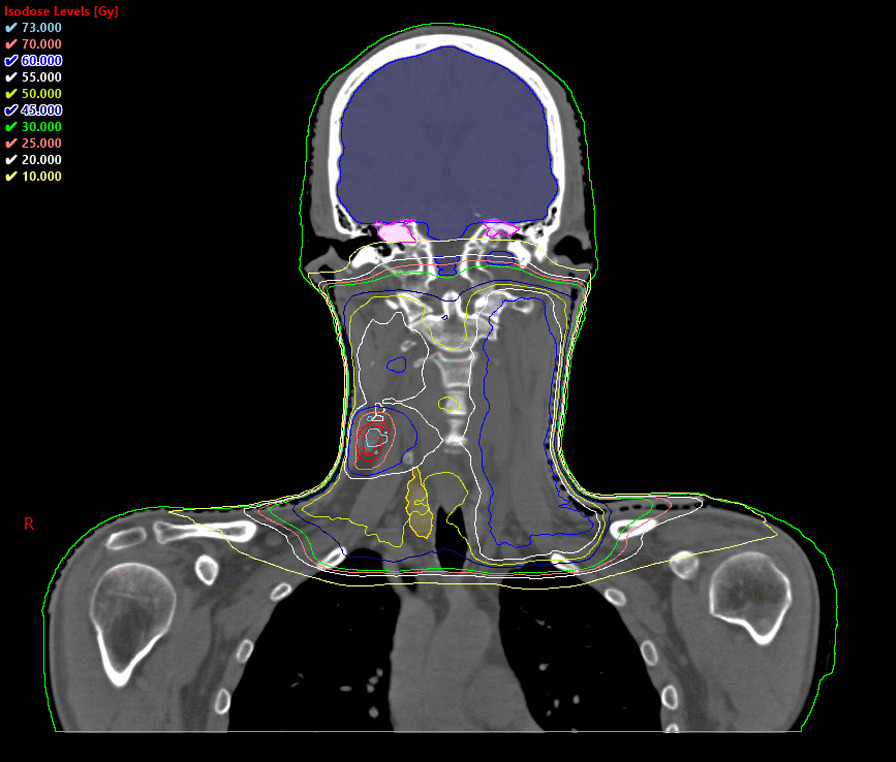
Dose distribution in the Eclipse system (physical dose sum of the conventional radiotherapy and the stereotactic radiosurgery boost). Isocenter plane of the stereotactic boost plan

The surgical procedure included excision of the left half of the tongue, the floor of the mouth and the sidewall of the throat and bilateral cervical lymph node dissection in groups I-V on the left side and I-III on the right side. The second part of the operation involved reconstruction by using an anterolateral thigh flap. After three days, reoperation was performed due to hemorrhage from the wound after lymphadenectomy. The final histopathology report revealed G2 keratinizing squamous cell carcinoma, stage IVa (pT3 pN2b, AJCC 8th edition). Additional risk factors included perineural invasion of small nerves, an unfavorable pattern of invasion with small islands (the worst pattern of invasion, 4), a closest margin below 1 mm, and five metastatic lymph nodes (of 49 lymph nodes dissected) without extracapsular extension at levels II, IV, V on the left side. The novel prognostic histopathological grading system in oral squamous cell carcinoma based on tumor budding revealed G-2 [4]. There were 102 budding foci detected per 10 high-power fields. Two to four cell-sized nests or single-cell invasion was observed. A multidisciplinary case conference qualified the patient for postoperative radiochemotherapy, but he refused systemic treatment. Given the patient’s decision, radiotherapy alone was recommended. Five-point head, neck and shoulder masks were employed for patient immobilization, and a CT scan (3 mm slice thickness) without intravenous contrast with the patient in the supine position was performed in the planning radiotherapy process. The dose prescription involved the bilateral lymph nodes I-V to a total dose of 50 Gy in 25 fractions, the left side neck in groups II-V to a total dose of 60 Gy in 30 fractions and the tumor bed with bilateral submandibular lymph nodes to a total dose of 66 Gy in 33 fractions (Fig. 1). The VMAT (volumetric modulated arc therapy) technique was applied. In the first stage of treatment, three full arcs were used, followed by three arcs lateralized to the left side of the neck in the second stage. In the third stage, two frontal arcs were applied. Radiotherapy was performed by linear accelerators (Clinac 23EX; Varian Medical Systems, Palo Alto, CA, USA) with an energy of 6 MV and maximal dose rate of 600 MU/min. Two weeks after the beginning of radiotherapy, ultrasound of the neck was performed due to a suspected lump in group III on the right side. It revealed a 16 × 9 mm lymph node. Fine-needle aspiration biopsy was performed in the next stage, confirming metastasis of the squamous cell carcinoma. The multidisciplinary case conference urged the application of a single-fraction stereotactic radiosurgery (SRS) boost of 18 Gy to the metastatic lymph node. After 29 days of conventional treatment, a new 5-point mask, a new CT scan without contrast and an MR scan with gadolinium intravenous contrast (both with 1 mm slice thickness) in the supine position were performed in the planned stereotactic boost process. The GTV_boost_ volume was 0.7 cm^3^. A 3 mm margin was added to the GTV_boost_ to create a PTV_boost_ volume of 3.1cm^3^. A new treatment plan consisting of four arcs was prepared. Four 6 MV FFF (flattening filter-free) photon beams with a maximal dose rate of 1400 MU/min were utilized. The dose that fully covers target volume was selected as the prescribed isodose level, so that the minimum dose in the target volume was 100% of the prescribed dose and the maximum dose was 108.4%. The angle range of arcs was adapted to the boost localization and was limited to the right and front sides of the patient. The boost was delivered two days after the last fraction of conventional radiotherapy with the same linear accelerator. Overall treatment time totaled 50 days. Figures 2 and 3 show the dose distribution of the SRS boost and the sum of conventional RTH with SRS boost, respectively. Dose analysis of the conventional and stereotactic radiotherapy is presented consecutively in Tables 1 and 2. During radiotherapy, oral mucositis and moist desquamation (grade 3, CTCAE v5.0) were observed in the irradiated area, which subsided within three months of follow up. Additionally, Staphylococcus aureus and Klebsiella oxytoca were identified from the throat culture in six week of conventional treatment, subsequently treated using antibiotic therapy consistent with the antibiogram (clindamycin, amoxicillin/clavulanate potassium). Blood tests showed grade 1 anemia and leukopenia after 24 and 33 fraction respectively, and normalized seven months after treatment. Three, ten and thirteen months after the end of radiotherapy, CT scans and laryngological examinations did not prove locoregional recurrence. Chest X-ray and abdominal ultrasound did not detect any metastatic changes one year after treatment. Xerostomia G2 was the only symptom of late toxicity fifteen months after radiotherapy.

**Table 1:** Conventional radiotherapy: dose analysis in target volumes and organs at risk

	Minimal dose (Gy)	Mean dose (Gy)	Maximal dose (Gy)
CTV_50_	47.51	62.76	70.41
PTV_50_	25.40	61.20	70.41
CTV_60_	58.05	65.54	70.41
PTV_60_	54.90	64.73	70.41
CTV_66_	63.68	67.22	70.41
PTV_66_	60.25	66.92	70.41
Lens right	1.81	2.04	2.22
Lens left	1.93	2.17	2.517
Eye right	1.54	2.12	2.81
Eye left	1.70	2.25	3.035
Cochlea right	2.84	5.47	10.64
Cochlea left	2.82	6.32	12.17
Chiasm	2.17	2.38	2.56
Optic nerve right	2.18	2.36	2.58
Optic nerve left	2.21	2.39	2.58
Brainstem	1.99	6.82	26.84
Brain	1.00	4.02	35.51
Mandible	3.90	46.55	66.14
Parotid gland right	3.22	32.72	65.03
Parotid gland left	5.67	32.75	65.65
Spinal canal	2.07	33.32	44.46
Larynx	45.53	53.41	67.44
Thyroid gland	26.84	51.91	61.66

**Table 2:** Stereotactic radiosurgery boost: dose analysis in target volumes and most important organs at risk (in other organs the maximal dose did not exceed 0.5 Gy)

	Minimal dose (Gy)	Mean dose (Gy)	Maximal dose (Gy)
GTV_boost_	18.23	19.11	19.51
PTV_boost_	18.00	18.88	19.52
Spinal canal	0.02	0.31	2.18
Larynx	0.06	1.47	7.44
Thyroid gland	0.03	0.44	9.91
Blood vessels	0.27	11.08	19.24

At 17 months of follow-up, the patient reported coughing, suffocation and bloody sputum.

A CT scan revealed infiltration (64 × 65 × 100 mm) in the 3rd segment of the right lung, encompassing the hilum and central part of the mediastinum. Pathological examination after endoscopic ultrasound-guided fine needle aspiration (EUS-FNA) proved squamous cell carcinoma with moderate PD-L1 membrane expression (< 50%). Acknowledgment of the tumor in the chest as lung cancer or metastatic disease due to oral cancer was difficult; nevertheless, a further aggressive course might suggest a new primary cancer. The patient was qualified for palliative radiotherapy to a total dose of 30 Gy in 10 fractions (VMAT technique, single arc, gantry angle 30.0–210.0 deg) for the tumor in the chest (systemic treatment was contraindicated in terms of performance status—ECOG 2). Three months later, multiple metastases in the mediastinum, right lung, spleen and bones were detected by PET/CT. The most painful osteolitic lesion in the left ischium and femoral head was irradiated in one fraction to 8 Gy (two-dimensional radiotherapy technique, two coaxial opposite beams from AP and PA directions were used). The patient died the next month.

## Discussion

The diagnosis of head and neck cancer is associated with unfavorable prognosis, especially in IV stage. Median survival time is particularly poor for oral cavity cancer (23 months) compared to other localizations in head and neck (e.g. 59 months for laryngeal cancer). Most common pattern of failure is locoregional recurrence concerning even 50% of patients. Rapid regional recurrence can be associated with tumor size, extranodal extension and perineural invasion [5–7]. Stage of disease and histopathological risk factors fall into the pattern of aggressive course and poor prognosis of our patient, effectively treated with using of high-dose stereotactic radiotherapy boost without locoregional relapse. Stereotactic body radiotherapy (SBRT) in the head and neck region is increasing in popularity, especially in recurrent cases. Roh reported 36 patients (44 sites) who were reirradiated due to locally recurrent head and neck cancer to a total dose of 18–40 Gy in 3–5 fractions with CyberKnife radiosurgery as salvage treatment. Eleven sites concerned lymph nodes (neck and retropharyngeal). Thirty-one of 44 sites were evaluated for response. The author suggested that stereotactic body radiotherapy could be an effective treatment for recurrent disease with relatively good tolerance (thirteen patients with acute complications, three patients with late complications including necrosis) [8]. Moreover, a meta-analysis including ten articles (575 patients) explored problems of reirradiation using SBRT in management of recurrent or second primary head and neck cancer not suitable for salvage surgery. Total doses ranged from 24 to 44 Gy (median, 30 Gy) realized in 3–6 fractions (median, 5 fractions). The pooled rate of 2-year OS and LC were 30.0% and 47.3%, respectively. Complete response rate got 31.3% of patients. In researchers’ view, severe toxicity rate (grade ≥ 3) was acceptable, below 10% [9]. Collected data by Vargo et al. from eight institutions in USA showed that using IMRT technique (≥ 40 Gy) for definitive reirradiation of unresectable, recurrent or second primary head and neck cancer gave 35.4% 2-year OS rate in comparison to 16.3% for SBRT (1–5 fractions of ≥ 5 Gy/fraction). In this paper, researchers took into account Recursive Partitioning Analysis (RPA) classification and demonstrated favorable OS in class II patients, who underwent treatment with IMRT. There were no differences in OS in class III regardless of applying IMRT or SBRT technique. Acute toxicity in grade ≥ 3 was about 5 percentages points higher in IMRT than SBRT. Late toxicity was similar in both groups [10]. Compared with salvage treatment, our case report provides an example of the utilization of SBRT in primary radiotherapy as a method of escalation of the total dose during conventional radiotherapy in RPA class I patient. Dutch researchers described an SBRT boost (3 × 5.5 Gy) delivered with a CyberKnife for 195 patients with T1-T3 oropharyngeal squamous cell carcinoma after 46 Gy IMRT (23 daily fractions, six fractions per week). Concurrent systemic treatment (cisplatin or cetuximab) was administered only in 6% of patients (stage T3 or N2c without contraindications for systemic treatment). The 2-year OS and DFS were 87% and 81%, respectively. Sixty-five patients required a feeding tube due to acute toxicity, and 47 patients developed grade ≥ 3 late toxicity. This paper confirms that a combination of SBRT and IMRT can lead to successful outcomes, but side effects were the most serious problem of this treatment. Most soft tissue necrosis appears during the 12 months after the completion of RTH [11]. In the same follow-up period, we did not detect any serious side effects associated with the SBRT boost. Our patient reported xerostomia G2, which was bound with an exceeded tolerance dose in the parotid glands during conventional treatment as a result of needing to deliver a prescribed dose to the oral cavity. Interesting results were presented by Sher et al. on a group of 29 patients with Tis to T2 glottic cancer who were treated with hypofractionated radiotherapy—4 patients to 50 Gy in 15 daily fractions, 13 patients to 45 Gy in 10 fractions (three fraction per week) and 12 patients to 42.5 Gy in 5 twice-weekly fractions. During a median follow-up of 39.2 months, 5 local failures (4 patients with primary T2 tumors and 1 patient with primary T1b tumors) were diagnosed: two in the 50 Gy/15 fractions group and three in the 45 Gy/10 fractions group. There were no local recurrences in the 42.5 Gy/5 fractions group. The authors also highlighted dose-limiting toxicity grade 3 dysphagia and grade 4 laryngeal edema in one patient who was treated to 42.5 Gy/5 fractions and one patient who was treated to 45 Gy/10 fractions. They suggested that the large target volumes (PTVs, 17 cm^3^ and 21.3 cm^3^) and active smoking were the causes of the elevated risk of radiation-induced toxicity [12]. Regarding our case report, the patient did not smoke, and the PTV volume was slightly greater than 3 cm^3^; thus, this could be the reason why the SBRT boost was well tolerated. The localization of the target volume should not be ignored; treatment of nodal metastases seems safer than that of a primary tumor of the mucosa or digestive or respiratory tract. Data from the literature indicate that using very high single fraction radiation doses (15–25 Gy) in a mouse model could generate strong CD8 + T cell-dependent immunity, leading to tumor reduction [13]. Due to the patient’s declining systemic therapy during radiotherapy, the stereotactic single fraction boost may have contributed to compensating for the lack of chemotherapy by stimulating the immune system. Curious results were obtained from a Czech study involving patients ineligible for surgical treatment with advanced-stage floor of the mouth cancer [14]. After radical radiotherapy to a total dose of 70–72.5 Gy in 35/50 fractions, a 10 Gy boost in two fractions was applied. The 5-year follow-up revealed 62% local control and 27% overall survival (secondary malignancy was the cause of death in 5% of cases) with good tolerance—acute mucositis G3 and dysphagia G3 in 10% of patients. In the context of our case report and previously published experience [15], involving adenoid cystic carcinoma of the choanae and nasopharynx successfully treated with an 18 Gy boost after conventional radiotherapy, we show that stereotactic boost might also be applied in one fraction with effective local control and acceptable tolerance. Unfortunately, the dynamic course of a second malignancy (lung cancer) led to death, similar to two cases in the Czech publication.

In conclusion, the reported case is an example of a modern and effective approach of radiation therapy alone in the radical treatment of head and neck cancer. The expected direction of the development should involve prospective trials with a large group of patients for evaluating the efficacy and toxicity of SBRT boosts combined with standard fractionation radiotherapy as well as with systemic therapy, with particular emphasis on immunotherapy (ongoing RTOG 3507 KEYSTROKE study with pembrolizumab), especially in the aspect of stimulation of immune system after the application of high-dose radiotherapy.

## Data Availability

The datasets used and analyzed during the current study are available from the corresponding author on reasonable request.

## Abbreviations

AP: Anterior–posterior
AJCC: The American Joint Committee on Cancer
CT: Computed tomography scan
CTCAE: Common terminology criteria for adverse events
CTV: Clinical target volume
DFS: Disease-free survival
ECOG: Eastern Cooperative Oncology Group
EUS-FNA: Endoscopic ultrasound-guided fine needle aspiration
FFF: Flattening filter‐free
GTV: Gross tumor volume
H&N: Head and neck
IMRT: Intensity modulated radiation therapy
LC: Local control
MR: Magnetic resonance
OS: Overall survival
PA: Posterior–anterior
PD-L1: Programmed death-ligand 1
PET/CT: Positron emission tomography–computed tomography
PTV: Planning target volume
RTH: Radiotherapy, radiation therapy
RPA: Recursive partitioning analysis
SBRT: Stereotactic body radiotherapy
SRS: Stereotactic radiosurgery
VMAT: Volumetric modulated arc therapy
